# Killer Ig-Like Receptors (KIRs): Their Role in NK Cell Modulation and Developments Leading to Their Clinical Exploitation

**DOI:** 10.3389/fimmu.2019.01179

**Published:** 2019-05-28

**Authors:** Daniela Pende, Michela Falco, Massimo Vitale, Claudia Cantoni, Chiara Vitale, Enrico Munari, Alice Bertaina, Francesca Moretta, Genny Del Zotto, Gabriella Pietra, Maria Cristina Mingari, Franco Locatelli, Lorenzo Moretta

**Affiliations:** ^1^Laboratory of Immunology, Department of Integrated Oncological Therapies, IRCCS Ospedale Policlinico San Martino, Genoa, Italy; ^2^Laboratory of Clinical and Experimental Immunology, Integrated Department of Services and Laboratories, IRCCS Istituto G. Gaslini, Genoa, Italy; ^3^Department of Experimental Medicine (DIMES), Center of Excellence for Biomedical Research, Università di Genova, Genoa, Italy; ^4^Department of Experimental Medicine (DIMES), Università di Genova, Genoa, Italy; ^5^Department of Pathology, IRCCS Sacro Cuore Don Calabria Hospital, Negrar, Italy; ^6^Division of Stem Cell Transplantation and Regenerative Medicine, Department of Pediatrics Stanford School of Medicine, Stanford, CA, United States; ^7^Department of Laboratory Medicine, IRCCS Sacro Cuore Don Calabria Hospital, Negrar, Italy; ^8^Core Facilities, Integrated Department of Services and Laboratories, IRCCS Istituto G. Gaslini, Genoa, Italy; ^9^Department of Oncohematology and Cell and Gene Therapy, IRCCS Ospedale Pediatrico Bambino Gesù, Rome, Italy; ^10^Laboratory of Tumor Immunology, Department of Immunology, IRCCS Ospedale Pediatrico Bambino Gesù, Rome, Italy

**Keywords:** HLA class I, killer immunoglobulin-like receptors, KIR ligands, inhibitory checkpoints, NK alloreactivity, NK cell education, polymorphism

## Abstract

Natural killer (NK) cells contribute to the first line of defense against viruses and to the control of tumor growth and metastasis spread. The discovery of HLA class I specific inhibitory receptors, primarily of killer Ig-like receptors (KIRs), and of activating receptors has been fundamental to unravel NK cell function and the molecular mechanisms of tumor cell killing. Stemmed from the seminal discoveries in early ‘90s, in which Alessandro Moretta was the major actor, an extraordinary amount of research on KIR specificity, genetics, polymorphism, and repertoire has followed. These basic notions on NK cells and their receptors have been successfully translated to clinical applications, primarily to the haploidentical hematopoietic stem cell transplantation to cure otherwise fatal leukemia in patients with no HLA compatible donors. The finding that NK cells may express the PD-1 inhibitory checkpoint, particularly in cancer patients, may allow understanding how anti-PD-1 therapy could function also in case of HLA class I^neg^ tumors, usually susceptible to NK-mediated killing. This, together with the synergy of therapeutic anti-checkpoint monoclonal antibodies, including those directed against NKG2A or KIRs, emerging in recent or ongoing studies, opened new solid perspectives in cancer therapy.

## Introduction

The groundbreaking discoveries made by Alessandro Moretta left a very big footprint in Immunology, and, more generally, in Medicine (1, 2). Indeed, also now that “the music is over” (3) the Alessandro's legacy will remain, witnessed by the many human lives saved thanks to his seminal studies and his continuous efforts to translate discoveries to the benefit of patients with leukemia or solid tumors.

Since the beginning of his scientific career, Alessandro focused his research on two different issues that revealed to be fundamental for his future major accomplishments: on one side, the generation of monoclonal antibodies (mAbs) to functional surface molecules expressed by human T cells, and on the other side, the development of a highly efficient T cell cloning technique allowing clonal growth of virtually 100% T cells (4). While these early studies provided important information on co-receptor molecules inducing accessory signals for T cell activation and on the frequency and subset distribution of T cells endowed with given functional capabilities, they offered an invaluable tool for the subsequent development of techniques suitable for efficient cloning of natural killer (NK) cells. In turn, NK cell clones were fundamental for the discovery of both killer immunoglobulin-like receptors (KIRs) and natural cytotoxicity receptors (NCRs), the two most important steps toward unraveling NK cell function.

### General View on NK Cells

Notably, although NK cells were discovered in the mid ‘70s, the molecular mechanism(s) by which they discriminate between tumor and healthy cells remained obscure for long time. In late ‘80s, the “missing self-hypothesis,” proposed by Karre and Ljunggren, inspired the subsequent seminal discoveries in mice and humans in early ‘90s (5). The “missing self-hypothesis” stemmed from the observation that NK cells could kill a lymphoma cell line that had lost major histocompatibility (MHC) class I surface molecules, while the original MHC class I^pos^ were resistant to lysis. A conceivable interpretation was that NK cells are able to sense the absence of “self” MHC class I molecules on target cells. Indeed, MHC class I specific inhibitory receptors were discovered in parallel in humans and mice (6, 7). Surprisingly, human and mice receptors were found to belong to distinct molecular families, namely the Ig-superfamily and the lectin family, respectively. The inhibitory signals generated by the engagement with their MHC class I ligands resulted in NK cell inactivation. The prototypes of human leukocytes (HLA) class I specific receptors were two highly homologous molecules (p58.1 and p58.2) (8, 9), expressed by partially overlapping NK cell subsets. They were shown to recognize allotypic determinants shared by the two main groups of HLA-C alleles. While KIRs are expressed at late stages of NK cell differentiation, more immature NK cells express another important inhibitory receptor, the CD94:NKG2A heterodimer, specific for HLA-E (10). CD94:NKG2A is the first HLA class I specific receptor to be expressed during NK cell differentiation. At a further differentiation stage, it may be co-expressed with KIR(s), while it is lost by the most mature NK cells which express KIR(s) only (11). Notably, the pool of mature NK cells expresses at least one inhibitory receptor for self-HLA class I (whether KIR or NKG2A), thus avoiding autoreactivity. The very few NK cells lacking self-reactive receptors are anergic. This status is acquired during NK cell maturation by a process referred to as NK cell “licensing” or “education” (12, 13). Although HLA class I specific inhibitory receptors were fitting with the “missing self-hypothesis,” the understanding of the actual molecular mechanism(s) regulating NK cell function was incomplete, as the “on” signals inducing NK cell activation in the process of tumor cell lysis were unknown.

Again, the use of NK cell clones and the generation and selection of mAbs capable of modulating NK cell function allowed the identification of three novel activating receptors: NKp46 (14, 15), NKp44 (16, 17), and NKp30 (18), collectively named NCRs (19), playing a major role in tumor cell recognition and killing. In addition, Alessandro identified the function of other surface molecules acting as co-receptors, including p75/AIRM1, IRp60, 2B4, NTBA, and NKp80 (20–25), and could also determine the activating function of DNAM-1 (CD226) and its corresponding ligands (namely PVR and Nectin-2) (26). It became evident that NK cell activation is dependent on an array of receptors and co-receptors that interact with ligands overexpressed or expressed *de novo* on “stressed” cells and on tumor-transformed or virus-infected cells. While, in an autologous environment, healthy cells express HLA class I molecules that generate inhibitory signals via KIR or NKG2A, tumor- or virus-infected cells may display HLA down-regulation, allowing NK cell triggering via activating receptors and consequent target cell killing. In the case of viral infections that do not down-regulate HLA class I, the susceptibility to NK-mediated killing may be related to viral peptides that, upon binding to HLA molecules, could impair KIR engagement.

Altogether, these findings revealed that NK cell activation is under the control of inhibitory and activating receptors and their ligands on target cells, and thus receptor/ligand pairs could represent true checkpoints in the regulation of NK cell function (27). Notably, an important mechanism of tumor escape is the down-regulation of activating NK receptor expression, thus eluding the NK-mediated control of tumor growth and metastatic spread (28–30).

In humans, two main NK cell subsets were originally identified on the basis of the intensity of CD56 surface expression. The two subsets are differently distributed in blood and tissues: CD56^dim^ are largely predominant in peripheral blood (PB), while CD56^bright^ are much more abundant in tissues. CD56^bright^ NK cells are relatively immature, express NKG2A and not KIR, are poorly cytolytic, secrete cytokines (primarily IFN-γ and TNF-α), and undergo intensive proliferation in response to IL-2 or IL-15. In contrast, CD56^dim^ NK cells express NKG2A and/or KIR, are mature, display a strong cytolytic activity and cytokine secretion capability rapidly upon activation. Remarkably, on the basis of the surface expression of NKG2A and/or KIR, and other markers, CD56^dim^ NK cells could be further subdivided in different subsets representative of distinct differentiation stages characterized by the progressive decrease of the proliferative capacity, paralleled by an increase of cytolytic activity (11, 31). The most mature, terminally differentiated, NK cells are KIR^pos^ CD57^pos^ CD16^bright^ and may express the HLA-E specific activating receptor NKG2C. As recently revealed (also with the Alessandro's contribution), NKG2C^pos^ cells undergo expansion in CMV infections, displaying adaptive features and memory-like function (32–35).

During the last decade, cells belonging to the innate lymphoid cells (ILCs) were identified. They share with NK cells a common ID2^pos^ lymphoid precursor. Absent or infrequent in PB of healthy individuals, they reside primarily in mucosal tissues, skin, and lymphoid organs (e.g., tonsils), where they participate to innate defense against pathogens and to tissue repair/regeneration (36–38). They are referred to as “helper” ILC, being non-cytolytic and producing typical sets of cytokines. While they will not be further discussed here, it is noteworthy that an important subset of ILC3 (the NCR^pos^ ILC3) is characterized by the expression of NCR, the activating receptors originally described and characterized by Alessandro.

NK cells can migrate from blood to tissues or lymphoid organs. Their traffic is regulated by chemokines and their corresponding receptors, addressing different NK subsets to specific compartments or inflammatory sites. In addition, since CD34^pos^ precursors, capable of differentiating toward NK cells, have been detected in tissues including liver (39), tonsils (40), thymus (41), and decidua (42), it is likely that some of the tissue resident NK cells may undergo differentiation from these precursors and, under the influence of specific tissue microenvironment, acquire unique functional properties.

While NK cells mediate a strong anti-tumor activity, their effectiveness may be greatly compromised by the suppressive microenvironment of different tumors. Suppression is mediated by a number of mechanisms, including release of soluble factors by tumor cells and by cells present in the microenvironment that have been attracted and/or “conditioned” by tumor cells. These cells include M2 macrophages, myeloid-derived suppressor cells (MDSC), T-reg and stromal cells (30). In addition, hypoxia, frequently occurring in tumor lesions, also contributes to the inhibition of immune effector cells. In the case of NK cells, the principal effect is the down-regulation of the activating NK receptors, thus rendering NK cells “disarmed” and unable to recognize specific ligands on tumor cells. Another related tumor-induced mechanism playing an important role in tumor escape is the expression of inhibitory checkpoints, primarily PD-1, in NK (and T) cells, and of the corresponding ligands in tumor cells (PD-L1 and PD-L2). The PD-1/PD-L1 interaction has been shown to induce NK cell inactivation (43). Notably, PD-1 expression in NK cells has been reported and functionally analyzed by Alessandro and coworkers (44). PD-1 will be briefly discussed, in the frame of novel immune-based strategies that appear very promising in tumor therapy. The important role of NK cells and their receptors in defense against tumors and leukemia is also witnessed by the great success achieved in the T-depleted, haploidentical hematopoietic stem cell transplantation (haplo-HSCT) setting to cure high-risk leukemia. The unthinkable benefit of this therapeutic approach is primarily related to the graft-vs.-leukemia effect of donor NK cells, arising from grafted stem cells and/or infused with the graft.

Thus, in less than 3 decades, a cell of the innate immunity that was substantially underscored (45) became an important tool to successfully treat otherwise incurable leukemia. Many of these major therapeutic achievements are based on seminal Alessandro's discoveries illustrated in this review.

While the main focus of our applied researches has been the involvement of KIRs in defense against tumors and leukemia, it is important to underline that KIRs and their polymorphisms have been shown to play a relevant role also in inflammation, infection, autoimmune diseases, and reproduction (46–48).

## Milestones in the Discovery of HLA Specific Receptors in Early ‘90s

In late ‘80s, Alessandro Moretta came to Genoa from Lausanne with a “very special cell line”: the hybridoma producing GL183 mAb. Through this mAb he identified a novel 58-kD surface molecule expressed on a subset of NK cells and capable of modulating NK cell cytolytic function (8). The paper describing this molecule was published in 1990 and this was the beginning of a new exciting era that led to the discovery of various HLA class I specific receptors, using the same approach based on mice immunization with NK cell clones and on the hybridoma technology. Soon thereafter, by immunizing mice with a GL183^neg^ NK cell clone, EB6 mAb was selected for its ability to react with a molecule distinct from GL183 but sharing most of its characteristics, as revealed by phenotypic, biochemical, and functional studies (9). Notably, the reactivity of the two mAbs allowed the identification of four NK cell subsets that could be extremely variable in terms of relative percentages among different individuals. Both mAbs could inhibit the cytolytic activity of selected NK clones (expressing either GL183 or EB6) in redirected killing assays, using the FcγR^pos^ murine P815 target cell line. Interestingly, a striking correlation existed between the EB6^pos^ GL183^neg^ phenotype of NK cell clones and their ability to lyse normal allogeneic cells, such as PHA-blasts, in a previously defined type of alloreactivity (i.e., stimulator/responder combination in mixed lymphocyte reaction, MLR), termed “1 anti-A” or “group 1” (49–51). In the same years the seminal study by Ljunggren and Karre (an elaboration of the Karre's PhD thesis in early ‘80s) was published, formulating the “missing self” hypothesis (5). In humans, p58 molecules appeared to fit with this proposal, rendering NK cells capable of self/non-self discrimination. Then a close relationship could be determined between the expression of EB6- and GL183-reactive molecules, termed p58.1 and p58.2, respectively, and their specificity for different HLA-C alleles (52–54). Evidence was provided that HLA-C^*^03 and HLA-C^*^04 conferred selective protection from GL183^pos^ and from EB6^pos^ NK clones respectively (52, 55). These two specificities were extended to a series of HLA-C alleles related to HLA-C^*^03, characterized by Ser^77^-Asn^80^, and those related to HLA-C^*^04, characterized by Asn^77^-Lys^80^ (56). These two distinct groups of HLA-C alleles encompass virtually all the expressed HLA-C molecules. Thus, EB6^pos^ GL183^neg^ “group 1” NK clones could lyse allogeneic cells expressing only HLA-C alleles with Ser^77^-Asn^80^ motif, while EB6^neg^ GL183^pos^ “group 2” NK clones were alloreactive against target cells expressing only HLA-C alleles with Asn^77^-Lys^80^ motif. Importantly, treatment of NK clones with mAbs masking p58 molecules resulted in the killing of HLA-C protected cells, showing that this inhibitory receptor/ligand interaction was responsible for the blocking of target cell lysis (55). Consistent with these notions, NK clones co-expressing p58.1 and p58.2 molecules were inhibited by the interaction with any allogeneic target cells, thus being unable of any type of alloreactivity (57). In the same years, NKB1 was described as a 70 kDa glycoprotein, whose expression was detected by DX9 mAb, and DX9^pos^ NK clones recognized a group of HLA-B alleles carrying the Bw4 public epitope (58, 59). Thus, analysis of NK cell clones, derived not only from different donors but also from single donors, allowed the detection of the great NK cell heterogeneity with extremely variable patterns of expression and co-expression of the various receptors with different HLA specificities. In 1992, studying in different donors the NK cell repertoire related to the ability to lyse allogeneic cells, five distinct allospecificities were described (60). While “group 1” and “group 2” had been previously defined, “group 3” could be later explained by the expression of p70 alone (detected by Z27 mAb), and “group 5” by the expression of both p58.1 and p70 (57). These data supported the notion that each HLA class I specific receptor could be physically and functionally independent (57, 61). Another important piece of history was added by the evidence that EB6 and GL183 mAb could also recognize activating receptors, named p50.1 and p50.2, respectively, according to their molecular mass (62). Subsequently, another activating receptor, termed p50.3, was identified by three PAX mAbs and was not stained by either GL183 or EB6 mAb (63). Differently from p58 molecules, found in all individuals, p50 receptors could be detected only in some donors. A crucial step was represented by the identification of the cDNAs coding for these receptors, demonstrating that they belong to the same molecular family (64). The p58 and p50 molecules were highly homologous in their extracellular regions formed by 2 Ig-like domains, while major differences existed in their transmembrane and cytoplasmic portions. Notably, while in p58 the transmembrane region included only non-polar residues, in p50 it contained the charged amino acid Lys. Moreover, whereas p58 displayed a 76–84 amino acid cytoplasmic tail containing two YxxL motifs (D/Ex_8_D/ExxYxxLx_26_YxxL), later referred to as immunoreceptor tyrosine-based inhibitory motifs (ITIMs), p50 was characterized by a shorter tail (39 amino acids) lacking YxxL (64–66). The cDNA encoding the Bw4 specific receptors, named NKB1/p70, was also identified, revealing that its extracellular region was composed by 3 Ig-like domains and that its cytoplasmic tail included ITIMs (65, 67). All the genes coding for these receptors were mapped on chromosome 19q13.4. Concomitantly, cloning of CD94-encoding gene revealed that CD94 is a type II protein of the C-type lectin superfamily and that this gene is located on chromosome 12 (68). NK cell clones expressing CD94^bright^ phenotype did not lyse cell lines transfected with different HLA class I alleles, and their cytotoxicity could be restored by mAb-mediated masking of CD94 or HLA class I (69). Moreover, a functional ambivalence of CD94-associated surface antigens was described (see below) (70). Finally, an additional inhibitory NK receptor of the Ig-like superfamily was identified to be specific for HLA-A^*^03 and -A^*^011 allotypes (71, 72). In our laboratory the production of Q66 mAb allowed the characterization of this receptor, which appeared to be a disulphide-linked dimer and was termed p140. Importantly, KIRs (and NKG2A) were also detected in a subset of CD8^pos^ αβ T cells (73) in which they could interfere with TCR-mediated cell activation. These T cells were found to represent oligoclonally or monoclonally expanded cell populations (74). Subsequent studies revealed that such KIR^pos^ CD8^pos^ cells were HLA-E restricted (75, 76).

All these data, published between 1990 and 1996, represent true milestones for our present knowledge of the HLA specific receptors. Alessandro pioneered these studies with the discovery of p58 receptors and gave a fundamental contribution to most of the other major findings. Tracking all the rational steps leading to these discoveries in a seminal Alessandro's review, published in Annual Review in Immunology in 1996, has been for us both moving and impressive (7). Indeed, his contributions still represent a complete and modern insight on NK cell biology.

## KIR Nomenclature

Different research groups started to work on the NK cell recognition of HLA class I molecules, therefore receptors and encoding cDNAs were named differently, so that the need for a common nomenclature arose. Thus, in late ‘90s, the acronym KIR (killer-cell inhibitory receptor) was proposed to indicate the members of this novel family (77). Then, following the experimental evidences that some KIR receptors were able to transduce activating signals, it was suggested to preserve the KIR acronym simply changing the meaning of “I” into “immunoglobulin-like.” The KIR nomenclature was designed in order to reflect the structure and the function of the encoded molecules, as well as the nucleotide sequence similarity among the different *KIR* family members. Thus, the first 2 digits following KIR correspond to the number of the extracellular domains (2D and 3D), while the third digit provides information on the length of the cytoplasmic tail (L or S) and consequently reveals the protein function (inhibitory or activating, respectively). Moreover, the two pseudogenes are indicated with the letter “P” (namely, *KIR2DP1* and *KIR3DP1*) (78). The establishment of a centralized, publicly accessible KIR database (IPD-KIR), collecting all the officially recognized nucleotide and protein sequences, has been of great help to all the immunologists involved in the study of *KIR* gene polymorphism (79). Temporally, the first KIR Nomenclature report coincided with the first release of the IPD-KIR database. Finally, like other immune cell surface proteins, a CD number has been assigned to KIR proteins (namely the CD158 series).

Reconnecting with what has been described in the previous paragraph, it is possible to attribute the KIR nomenclature to the molecules with the “old” denomination. Thus, p58.1 and p50.1 correspond to KIR2DL1 and KIR2DS1, p58.2 and p50.2 to KIR2DL2/L3 and KIR2DS2, p50.3 to KIR2DS4, NKB1/p70 to KIR3DL1, and p140/NKAT4 to KIR3DL2.

## *KIR* Genes Display an Extraordinary Level of Polymorphism

*KIR* gene family is characterized by an extraordinary high degree of diversity, which arises from variability in *KIR* gene content, due to differences in both presence/absence (P/A) of *KIR* genes and *KIR* gene copy number, and from allelic polymorphism (80–89). Several strategies have been developed to analyze *KIR* repertoires, including approaches able to analyze P/A of the different *KIR* genes, *KIR* gene copy number variations, and, more recently, a “whole” *KIR* allele typing by next generation sequencing (NGS) approach (87, 90–100) underlining the huge interest of the immunologists in the KIR field.

### *KIR* Haplotypes

**Figure 1:**
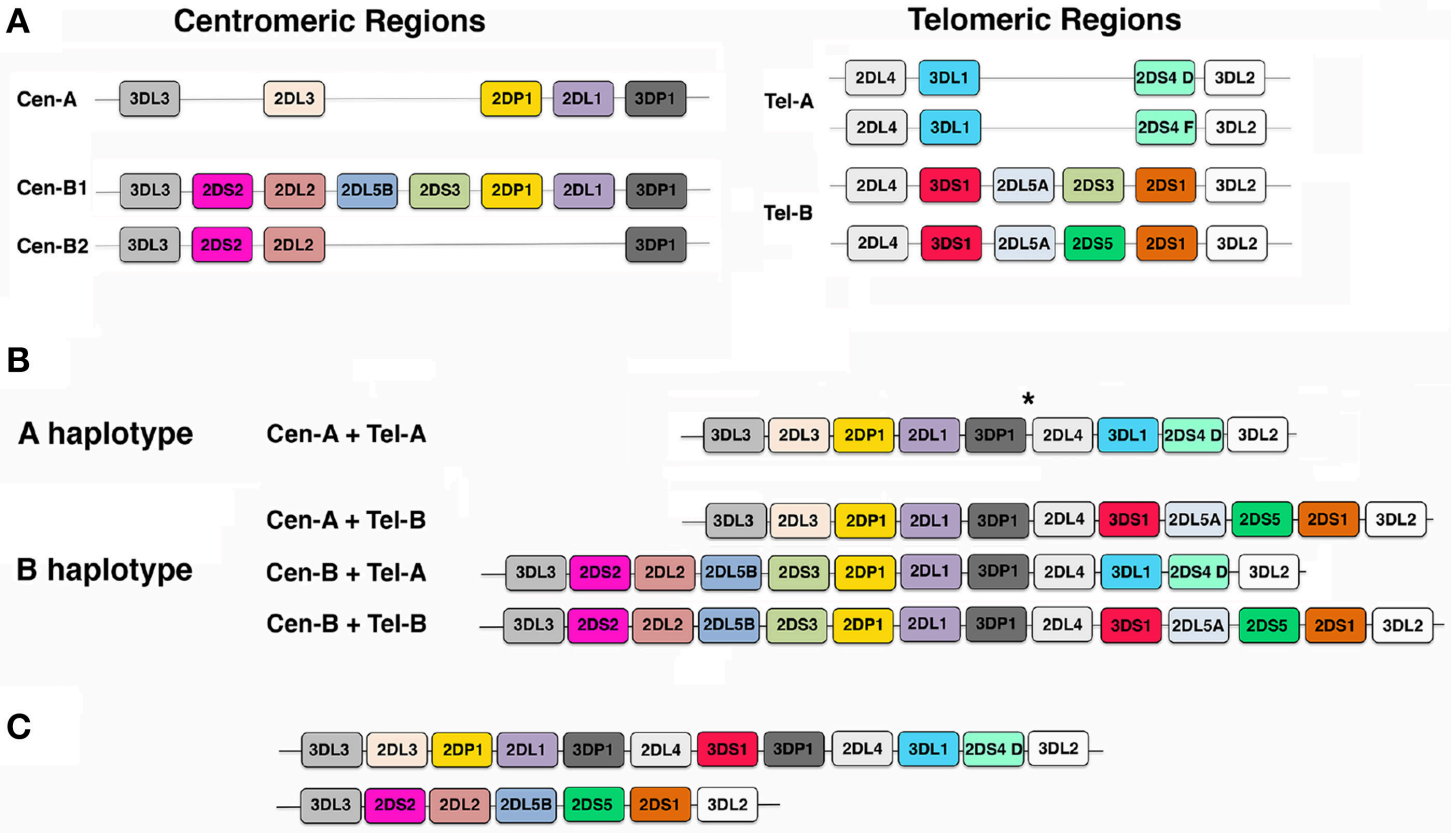
Gene organization of *KIR* locus. **(A)** A cartoon representation of the most frequent centromeric (Cen) and telomeric (Tel) regions detected in Caucasians. **(B)** Schematic pictures of *KIR* gene order in the A haplotypes and in 3 representative B haplotypes. ^*^ indicates the hot spot of recombination. **(C)** Examples of an extended and a truncated haplotype. Each colored box represents a *KIR* gene; for simplicity *KIR* gene names are reported in the boxes without the *KIR* acronym.

As previously mentioned, *KIR* gene family has been mapped on chromosome 19q13.4 where *KIR* genes, each spanning ~10–16 kb, are tightly arranged in a head-to-tail orientation. This family consists of 13 genes (*KIR2DL1, KIR2DL2/L3, KIR2DL4, KIR2DL5A, KIR2DL5B, KIR2DS1, KIR2DS2, KIR2DS3, KIR2DS4, KIR2DS5, KIR3DL1/S1, KIR3DL2, KIR3DL3*) and 2 pseudogenes (*KIR2DP1*, and *KIR3DP1*) (82, 101, 102). With few exceptions, *KIR* haplotypes can be divided in two regions, termed centromeric (Cen) and telomeric (Tel), by the presence of a recombination hot spot (86, 87, 89). Four genes, named framework genes (i.e., *KIR3DL3, KIR3DP1, KIR2DL4*, and *KIR3DL2*), mark the beginning and the end of these two regions. Based on the different number and kind of *KIR* genes present, three different centromeric regions (referred to as Cen-A, Cen-B1, and Cen-B2) and two different telomeric regions (namely Tel-A and Tel-B) have been identified (Figure 1A). Despite the high level of *KIR* gene content variability described, two main groups of *KIR* haplotypes, termed as “A” and “B,” have been identified (46, 80, 87, 102–106). The A haplotypes have a fixed gene content and are composed by Cen-A and Tel-A regions (Figures 1A,B). They include inhibitory *KIR* genes coding for receptors recognizing HLA-C (*KIR2DL1* and *KIR2DL3*), HLA-B (*KIR3DL1*), HLA-A (*KIR3DL2*), and HLA-G (*KIR2DL4*) molecules and only one activating *KIR* (*KIR2DS4*) that, in some A haplotypes, does not code for a surface receptor (102). The remaining *KIR* haplotypes are collectively referred to as B haplotypes, and comprise haplotypes carrying Cen-A/Tel-B, Cen-B/Tel-A, and Cen-B/Tel-B combinations (Figure 1B). They are characterized by the presence of at least one of the following *KIR* genes: *KIR2DS2, KIR2DL2, KIR2DL5B, KIR2DS3, KIR3DS1, KIR2DL5A, KIR2DS5*, and *KIR2DS1*. Consequently, *KIR* A haplotypes differ from each other predominantly on the basis of *KIR* allelic polymorphism, while *KIR* B haplotypes mainly by their gene content. Notably, although with different frequencies, A and B haplotypes are maintained within all human populations, strongly suggesting that they have distinct and complementary functions and that they are under balancing selection (46, 107, 108). Moreover, the peculiar structure of *KIR* locus, including the presence of several genes sharing exon/intron organization, having a high degree of homology, and displaying the same orientation, facilitates non-reciprocal recombination that promotes deletion or duplication of *KIR* genes (i.e., formation of truncated or extended haplotypes), as well as the generation of hybrid *KIR* genes (i.e., *KIR* alleles including exons derived by different *KIR* genes) (Figure 1C) (86, 87, 109–113).

### *KIR* Allelic Polymorphism

As previously reported, the first *KIR* cDNA sequences have been cloned in mid-90s. The great interest in the *KIR* field led to the identification, at the present, of ~1,000 alleles (https://www.ebi.ac.uk/ipd/kir/). This extremely high level of polymorphism, second only to that of *HLA* genes, arose from studies including different populations and relatively simple methodologies. Several allelic polymorphisms have been reported to impact KIR protein expression and function. Indeed, the presence of particular amino acid residues determining protein misfolding and resulting in the lack of surface receptor expression has been identified among KIR3DL1, KIR2DL1, and KIR2DL2 allotypes (95, 114–117). Moreover, *KIR* alleles characterized by a termination codon causing the premature end of the protein (namely *KIR* Null alleles) have been reported. Additionally, some *KIR2DL4* and *KIR2DS4* alleles are characterized by nucleotide deletions that, causing frameshift, lead to truncated polypeptides (102, 118, 119). Notably, different clades of alleles coding for surface receptors characterized by a low or high surface expression have been detected in *KIR3DL1* and *KIR2DL1* genes (95, 120, 121). Polymorphisms affecting KIR function may also cause differences in ligand affinity or in signal transduction. In this regard, it is well-established that KIR2DL1 allotypes coded by Cen-A regions are stronger receptors than those coded by Cen-B1 regions, and KIR2DL2 allotypes (coded by Cen-B regions) have a higher ligand affinity than KIR2DL3 allotypes (coded by Cen-A regions) (116, 122). In addition, the amino acid polymorphisms of KIR3DL1, the KIR receptor displaying the highest level of allelic variation, have been shown to impact not only its surface expression but also its affinity for HLA class I Bw4 bearing allotypes (123–126). Different amino acidic variations relevant for KIR signal transduction have been also reported in the exons coding for the transmembrane and cytoplasmic regions. In particular, the lack of charged residues, important for its association with signal-transducing polypeptides, has been described for a KIR2DS2 allele (127). In *KIR2DL1*, polymorphisms in the exons of the cytoplasmic tail, either causing the premature termination of translation at the end of the transmembrane domain or affecting the strength of inhibitory signal, have been reported (128, 129).

Thus, the extraordinary plasticity of the *KIR* gene family and the variability due to both *KIR* gene content and allelic polymorphism make unlikely that unrelated individuals share the same *KIR* genotype.

## KIR/KIR-Ligand Pairs

As mentioned above, the HLA class I ligands recognized by KIR2DL1, KIR2DL2/L3, KIR3DL1, and KIR3DL2 have been identified almost conjointly with the characterization of the receptors. Thereafter, the resolution of the crystal structures of KIRs in complex with their ligands, the development of several tools including soluble KIRs, KIR tetramers, HLA class I tetramers folded with selected peptides, and retrovirally transduced human cell lines (i.e., NKL, Jurkat) expressing only one KIR, allowed to refine KIR/KIR-ligand interactions (122, 130–134).

### Inhibitory KIRs

**Figure 2:**
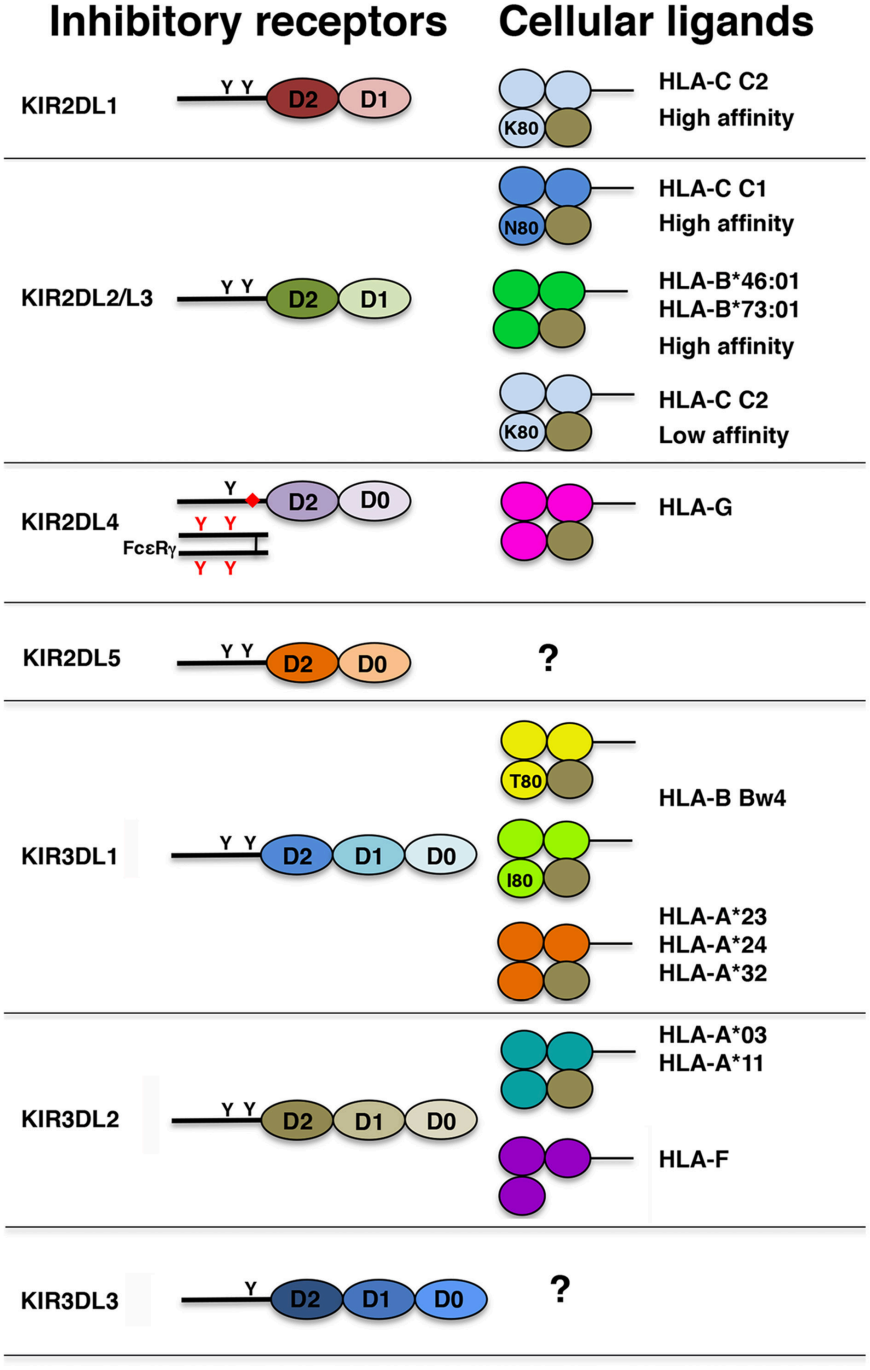
Schematic representation of the structure of iKIRs and their HLA class I ligands. Each Ig domain is depicted as an oval and, according to its structural features, labeled as D0, D1, and D2. Presence of ITIMs in KIR cytoplasmic tails and ITAMs in FcεR γ chain is indicated with black and red Y, respectively. The charged residue in KIR2DL4 transmembrane region is reported as a red diamond. Each domain of HLA class I molecules is represented as a colored circle and β2m as a brown circle. In HLA-C and HLA-B molecules, K80, N80, T80, and I80 indicate the presence, at residue 80, of lysine, asparagine, threonine, and isoleucine, respectively.

In Figure 2, the molecular structure of the inhibitory KIRs (iKIRs) and of their known cellular ligands is summarized. Using KIR2DL1-Fc (i.e., chimeric molecules comprising the extracellular portion of the receptor linked to the Fc region of human IgG1) in multiplex HLA class I binding assays, it has been possible to demonstrate that KIR2DL1 binds specifically, and with high avidity, to all HLA-C C2 but not HLA-C C1 or any HLA-A or HLA-B allotypes. The only exception is represented by KIR2DL1^*^022, a peculiar KIR2DL1 receptor carrying Lys^44^ (instead of Met^44^) that switches its ligand specificity to HLA-C C1 (129). By the use of KIR2DL1-Fc and plasmon resonance, it has been shown that several KIR2DL1 molecules recognize distinct HLA-C C2 allotypes with different avidities (the highest for HLA-C^*^15:02 and the lowest for HLA-C^*^04:01). As reported in early studies, and as expected on the basis of the homology in their extracellular domains, KIR2DL2 and KIR2DL3 bind a similar set of HLA-C ligands. Again, using KIR-Fc molecules it has been confirmed that KIR2DL2 and KIR2DL3 allotypes recognize, at high affinity, HLA-C C1 bearing epitope, and it has been demonstrated their ability to bind to two HLA-B allotypes (i.e., HLA-B^*^46:01 and HLA-B^*^73:01) that, being originated by crossing-over between HLA-C and HLA-B alleles, conserve the C1 epitope. Moreover, KIR2DL2 and KIR2DL3 display the capability to bind HLA-C C2 allotypes, even though with low affinity. Thus, in contrast to the simplicity of KIR2DL1 ligand recognition, a more complicated situation governs KIR2DL2 and KIR2DL3 ligand interactions (116, 122). The only known exception is represented by KIR2DL3^*^005 that, differing from other KIR2DL3 receptors, displays a HLA-C binding ability similar to that observed for KIR2DL2 allelic products (135). Increasing evidences indicate that both KIR2DL2/L3 and KIR2DL1, in addition to their ability to discriminate between C1 and C2 epitopes, bind numerous peptide/HLA-C combinations retaining a degree of peptide selectivity (136). Generally, KIR2DL2 and KIR2DL3 are characterized by a greater selectivity for peptide than KIR2DL1, and this ability seems to be particularly relevant for the recognition of the low affinity HLA-C C2 allotypes. Notably, the capability of KIR2DL1 to recognize HLA-C^*^08:02 (i.e., a HLA-C C1 allotype) presenting a restricted number of peptides has been recently reported (137). Taken together these studies underline how NK cells are able to sense, not only the down-regulation of HLA class I, but also alterations in HLA presented peptidome that may occur during viral infections or malignant transformations.

Between the two D0-D2 receptors, KIR2DL4 is the only one for which the ligand has been characterized. Differently from other iKIR2D receptors, KIR2DL4 binds to HLA-G, a non-classical HLA class I molecule that, in healthy cells, is restricted to the trophoblast cells invading the maternal decidua during early pregnancy. This receptor contains only one ITIM in the long cytoplasmic tail and a charged residue in the transmembrane region allowing its association with the γ chain of FcεR. These features render this receptor a peculiar member of the KIR family displaying an unusual hybrid structure and sharing characteristics with both inhibitory and activating KIRs. Functional studies revealed that KIR2DL4 is characterized by a weak inhibitory potential and that its engagement with soluble ligand results in strong cytokine release in the absence of killing (138, 139).

It is well-established that KIR3DL1 binds not only to HLA-B but also to some HLA-A Bw4 bearing allotypes. Indeed, it has been reported that among Bw4^pos^ HLA-A allotypes, HLA-A^*^24:02, -A^*^32:01, -A^*^23:01, but not HLA-A^*^25:01, are able not only to inhibit but also to educate KIR3DL1^pos^ NK cells (140). Among the five residues of α1 helix defining the Bw4 motif, the Ile/Thr dimorphism at residue 80 has been proposed as a marker of KIR3DL1 ligand affinity. Recent studies clearly indicate that, although many high affinity ligands possess Ile^80^ (Bw4 I^80^), KIR3DL1/HLA Bw4^pos^ allotype interactions are very complex and strongly dependent on the avidity and surface expression of both receptor and ligand. Indeed, functional analyses provided clear evidences that both KIR3DL1 polymorphism and ligand variability strongly affect the avidity of KIR/KIR-L combinations sharply influencing NK cell function (123, 126, 141). As previously mentioned, KIR3DL2 allotypes bind two HLA-A allotypes (HLA-A^*^03 and -A^*^11) (71, 72). Functional analyses revealed that this receptor is characterized by low inhibitory capacity and its ligand interaction is highly dependent on the HLA-A bound peptide (132). Recently, it has been demonstrated that KIR3DL2 also recognizes the non-classical HLA class I molecule HLA-F at open conformation (142). Another relevant function of this iKIR is represented by its ability to function as sensor for pathogen-associated molecular patterns. Indeed, KIR3DL2 has been shown to bind CpG oligodeoxynucleotides (ODNs), and its D0 domain is primarily involved in ODN recognition (143). Remarkably, interaction between KIR3DL2 and ODN did not result in the delivery of inhibitory signals but in a sharp down-modulation of its surface expression, and in the induction of cytokine release underlining the antimicrobial role of NK cells in the course of infection.

### Activating KIRs

**Figure 3:**
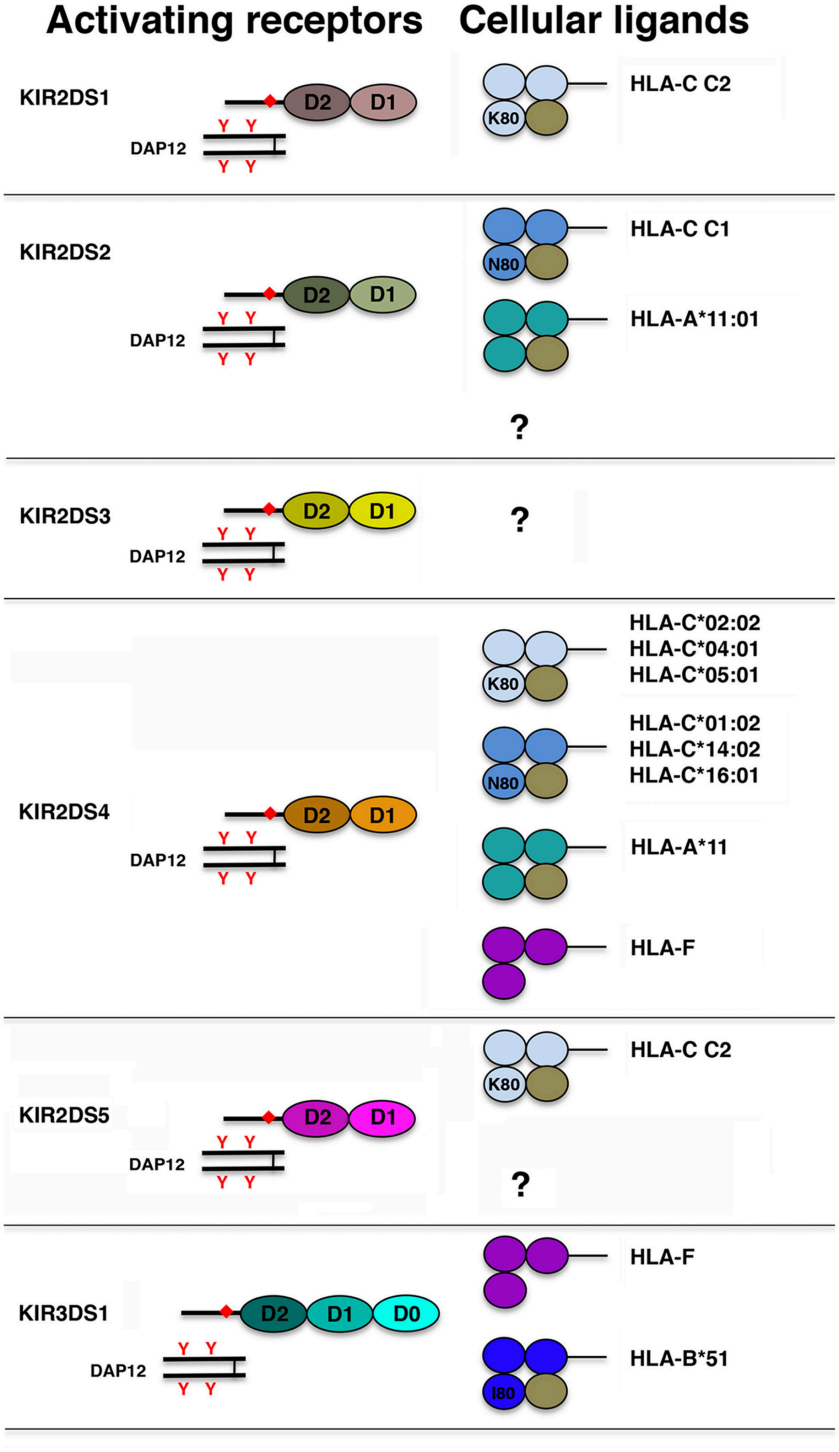
Schematic representation of the structure of aKIR and their HLA class I ligand. Each Ig domain is depicted as an oval and, according to its structural features, labeled as D0, D1, and D2. Presence of a charged residue in the KIR transmembrane region and of ITAMs in DAP12 cytoplasmic tail is indicated with orange diamond and orange Y, respectively. Each domain of HLA class I molecules is represented as a colored circle and β2m as a brown circle. In HLA-C and HLA-B molecules, K80, N80, and I80 indicate the presence, at residue 80, of lysine, asparagine, and isoleucine, respectively.

From a structural point of view, in addition to a short cytoplasmic tail lacking ITIMs, a common feature of activating KIR (aKIRs) is the presence, in their trasmembrane region, of a charged residue (Lys) that allows their association with the signaling adaptor protein KARAP/DAP12 containing immunoreceptor tyrosine-based activating motifs (ITAMs) (144, 145). With the exception of KIR2DS1, the aKIR ligands remained elusive for a long time. Indeed, although the extracellular domains of some aKIRs display a high sequence homology with those of some iKIRs (namely, KIR2DS1-KIR2DL1, KIR2DS2-KIR2DL2, and KIR3DS1-KIR3DL1 pairs), it has been difficult to demonstrate aKIR/HLA class I interactions. Figure 3 shows the different aKIR/ligand pairs identified so far.

The best characterized aKIR is KIR2DS1. Functional analyses using *in vitro* expanded KIR2DS1^pos^ NK cell clones, as well as KIR2DS1 soluble receptor and KIR2DS1 tetramer binding analyses, clearly demonstrated that this aKIR recognizes HLA-C C2 allotypes, although with an affinity lower than that of KIR2DL1 (i.e., its inhibitory counterpart) (62, 129, 146–149). Moreover, using both site-directed mutagenesis approaches and soluble receptors, it has been established that the residue at position 70 (Lys^70^ or Arg^70^) sharply affects the ligand affinity of KIR2DS1 molecules (146). Notably, a recent paper reported that modulation of HLA-C by certain strains of HCMV is required for a potent KIR2DS1-mediated NK cell activation. This strongly suggests a role of this aKIR in viral recognition (150). The HLA class I ligands recognized by KIR2DS2 have been identified only recently, and they include HLA-C C1 allotypes and HLA-A^*^11:01 (133, 147, 151). Remarkably, this aKIR/KIR-L interaction is highly peptide-dependent. In particular, the capability of KIR2DS2 to directly recognize peptides from HCV helicase presented by HLA-C^*^01:02 (an HLA-C C1 allotype) may explain, at least in part, the protective effect of this aKIR in chronic hepatitis C infection (152). In addition, it has been also reported that KIR2DS2 recognizes, on several human carcinoma cell lines, a still undefined, β2m-independent cell surface protein (153). Correlative studies reported that HIV-infected individuals carrying both KIR3DS1 and HLA-B Bw4 I^80^ display slower progression to AIDS and lower viral load. While these data could suggest KIR3DS1 interaction with HLA-B Bw4 I^80^ allotypes, the KIR3DS1 ligands have remained indefinite until recently, and they are represented by the open conformation of the non-classical HLA-F molecule and by the HLA-B^*^51, an HLA-B Bw4 I^80^ allotype (154–156).

KIR2DS3, KIR2DS4, and KIR2DS5 have no inhibitory counterparts. Most likely because KIR2DS3 does not seem to be expressed at the cell surface (157), it is still a receptor with unknown function and unknown ligand. On the contrary, the majority of KIR2DS4 and KIR2DS5 alleles code for surface activating receptors (63, 158). KIR2DS4 binds a minority of HLA-C allotypes carrying either C1 or C2 epitopes as well as HLA-A^*^11 (159). Recently, a highly peptide dependent ligand recognition has been reported also for KIR2DS4. This aKIR has been shown to recognize HLA-C^*^05:01 presenting a peptide derived from the highly conserved bacterial recombinase A, thus providing evidence that it is involved in defense against bacterial infections (160).

As previously reported, KIR2DS5 can be located either in the centromeric half of some KIR haplotypes (i.e., Cen-B1 region) or in the telomeric half (namely Tel-B region). With the only exception of KIR2DS5^*^005, KIR2DS5 centromeric alleles differ from the telomeric ones. Notably, four out of five KIR2DS5 centromeric alleles but only two out of six KIR2DS5 telomeric alleles code for receptors recognizing several HLA-C C2 molecules (161).

## NK Cell Education and Receptor Repertoire

During their development, NK cells go through a process termed “education,” involving the interaction between inhibitory NK receptor (iNKR) and self HLA class I molecules. Although different models have been proposed, a general rule for education is that the strength of inhibitory interaction(s) dictates the efficiency of NK effector function (12, 13, 162, 163). A prominent role is played by the highly diversified system represented by the combinations between inhibitory KIR with their KIR-L (iKIR/KIR-L pairs). Another relevant iNKR is represented by the CD94:NKG2A heterodimer, a type II protein belonging to the C-type lectin superfamily, which recognizes the non-classical HLA class I molecule HLA-E (10). Since HLA-E binds peptides cleaved from the leader sequences (from −22 to −14 residues) of HLA-A, -B, or -C, it has been generally considered as a sensor of the overall amount of HLA class I molecules expressed on the cell surface. Recently, the Met/Thr (M/T) dimorphism at position −21 of the leader sequence of HLA-B (i.e., −21M and −21T) has been described to strongly impact the CD94:NKG2A/HLA-E interaction (164). Indeed, only a minority of HLA-B allotypes having −21M can supply HLA-E binding peptides, while the majority carrying −21T do not. Accordingly, individuals with −21M HLA-B could show higher HLA-E expression and more efficient NKG2A^pos^ NK cells. The activating counterpart of CD94:NKG2A is represented by CD94:NKG2C heterodimer, which binds HLA-E as well, although with lower affinity.

Extremely variegated NK cell receptor repertoires can be observed among different individuals (7, 60, 80, 164–167). This diversity is primarily due to the high polymorphism of *KIR* and *HLA* class I genes, which segregate independently, leading to diverse compound genotypes (46). Another important feature of the repertoire is the clonal distribution of KIR and CD94:NKG2A. During education NK cells must engage at least one iNKR with cognate HLA class I to become fully functional, otherwise, they will be hypo-responsive. This process ensures that each NK cell produced in the body maintains tolerance to self. The hypothesis that NKR expression represents the result of a stochastic event, in which the various encoding genes are independently regulated, had been already discussed in Alessandro's review (7). From studies of NK cells at the clonal level, it was clear that each NK cell could express either one or more iNKR specific for self HLA (self-iNKR) as well as additional iNKR specific for non-self HLA. The presence of NK cells expressing a single self-iNKR confers an advantage to the host, since it allows to sense the loss or peptide-induced alteration of even a single HLA class I allotype, and thus to kill the damaged cell, through the mechanism of “missing self-recognition.”

While a strong interaction between iKIR and its KIR-L positively impacts NK cell education, the behavior of aKIR is opposite and induces down-regulation of NK cell responsiveness in the presence of a strong cognate ligand. This phenomenon has been described for KIR2DS1 and KIR3DS1. Thus, KIR2DS1^pos^ NK cells are anergic in HLA-C C2/C2 individuals, while they are educated in C1/Cx ones (168). Similarly, KIR3DS1-mediated positive recognition of HLA-B^*^51 (Bw4 I^80^) could be detected in NK cell clones derived from Bw4 I^80^ negative donors, but not in those from Bw4 I^80^ positive donors (156).

Although genetics of *KIR* and *HLA* has the major impact on the receptor repertoire of the circulating NK cell pool, also environmental factors may play an important role. In this regard, the phenotypic analysis of NK cells derived from twins, who are genetically identical for *KIR* and *HLA* genes, has been particularly relevant (169). Indeed, twins' NK cell populations are similar but not identical to each other, especially in adult age. In this context, viral infections may impact the NK cell phenotype. In particular, HCMV shapes the receptor repertoire, driving to adaptive NK cell differentiation through epigenetic alterations (170–173). Thus, the expanded NK cell subset is characterized by the expression of CD94:NKG2C, mainly co-expressing KIR2DL specific for self HLA-C allotypes, and CD57, a marker of terminally differentiated stage (32, 33). Recent data revealed that the expansion and differentiation of adaptive NKG2C^pos^ NK cells could be determined by differentially recognition of distinct HCMV strains encoding variable UL40 peptides (174).

Nowadays, new technologies allow the identification of distinct subsets by the detection of multiple molecules in the whole NK cell population, even freshly derived from blood. Multi-parametric flow cytometry makes it possible the simultaneous use of numerous mAbs, detecting the pattern of different subpopulations carrying one or another iKIR, aKIR, CD94:NKG2A, and CD94:NKG2C. Table 1 describes the reactivity of some anti-KIR mAb, that can be used in combination to dissect various iKIR and aKIR. Moreover, mass cytometry by time-of-flight (CyTOF) technology allows the concomitant assessment of more than 30 parameters, revealing that at least 30.000 and even more distinct PB NK phenotypes can be detected in each individual (169).

**Table 1:** Some monoclonal antibodies recognizing KIR.

**Clone**	**Specificity**	**Isotype**	**Source^§^**
143211	KIR2DL1/S5	IgG1	R&D
EB6B	KIR2DL1/S1, KIR2DL3*005	IgG1	Our lab, Beckman Coulter
11PB6	KIR2DL1/S1, KIR2DL3*005	IgG1	Our lab, Miltenyi Biotec
HP-3E4	KIR2DL1/S1/S4	IgM	BD Biosciences
HP-MA4	KIR2DL1/S1/S3/S5	IgG2b	Biolegend, eBiosciences
GL183	KIR2DL2/L3/S2	IgG1	Our lab, Beckman Coulter
Y249	KIR2DL2/L3/S2	IgM	Our lab
CH-L	KIR2DL2/L3/S2	IgG2b	BD Biosciences
DX27	KIR2DL2/L3/S2	IgG2a	Biolegend, Miltenyi Biotec
ECM-41	KIR2DL3 (not ^*^005 and ^*^015)	IgM	Our lab
180701	KIR2DL3 (not ^*^005 and ^*^015)	IgG2a	R&D
1F12	KIR2DL3/S2	IgG2b	C. Retière ^∧^
FES172	KIR2DS4	IgG2a	Our lab, Beckman Coulter
PAX180	KIR2DS4	IgG1	Our lab
UP-R1	KIR2DL5	IgG1	Biolegend
DF200	KIR2DL1/L2/L3/S1/S2/S5	IgG1	Our lab
NKVSF1	Pan KIR2D	IgG1	Miltenyi Biotec
Z27	KIR3DL1/S1	IgG1	Our lab, Beckman Coulter
DX9	KIR3DL1	IgG1	Miltenyi Biotec
Q66	KIR3DL2	IgM	Our lab
AZ158	KIR3DL1/S1/L2	IgG2a	Our lab

§ *The names of the vendor and/or the lab of production is indicated. “Our lab” means the laboratories directed by A. Moretta, L. Moretta and M.C. Mingari*.

∧ *C. Retiére: Etablissement Français du Sang-Pays de la Loire, Nantes, France*.

NK cell education in pregnancy or in disease and the particular KIR/HLA combinations associated with either a protective role or a detrimental effect in placentation, infectious diseases, autoimmune and inflammatory diseases, and in cancer have recently been reviewed (46–48, 136, 175). Our group studied NK cell education in disease, particularly focusing on leukemia patients receiving HSCT (see below) or patients with X-linked lymphoproliferative disease 1 (XLP1). XLP1 is a primary immunodeficiency caused by mutations in *SH2D1A*, the gene encoding the signaling lymphocyte activation molecule (SLAM)-associated protein (SAP). Seminal studies coordinated by Alessandro provided evidence that, in the absence of SAP, 2B4 and NTB-A (belonging to SLAM family) co-receptors associate with protein tyrosine phosphatases thus delivering inhibitory instead of activating signals (22, 24, 176). This specific immune dysfunction in XLP1 patients causes the inability of NK cells to kill EBV-infected B cells (B-EBV), over-expressing their ligands (i.e., CD48 and NTB-A), with dramatic clinical consequences. Following this knowledge, we further analyzed the NK cell receptor repertoire of these patients, showing that substantial proportions of NK cells lacking any inhibitory receptor specific for self-HLA (self-NKR^neg^) are present and are fully functional, indicating that inhibitory 2B4 participates to NK cell education (177). Remarkably, self-NKR^neg^ NK cells can efficiently kill CD48^neg^ target cells, such as mature DC, with consequently defective antigen presentation, further exacerbating the immune defect of these patients.

## Inhibitory Checkpoints in NK Cells and Their Targeting in Tumor Therapy

As previously reported, the HLA class I specific inhibitory receptors are constitutively expressed by NK cells and finely regulate their function preventing NK-mediated damage to healthy tissues and allowing the elimination of tumors displaying defective HLA class I expression. Additional inhibitory checkpoints are inducible and play a wider physiological role in immune cell homeostasis. On the other hand, in tumors, they may severely compromise NK-mediated responses and promote tumor growth and metastasis. In particular, CTLA-4 and PD-1 are major regulators of immune responses and contribute to the maintenance of peripheral T cell tolerance. PD-1, a member of the Ig-superfamily, was first identified in T lymphocytes (178) and, more recently, in NK cells (44). In cancer, upon binding to its ligands (PD-L1 and/or PD-L2) expressed on tumor cells, PD-1 may impair the antitumor cytotoxicity, thus favoring tumor immune escape. Human PD-1^bright^ NK cells have been detected in patients with CMV infections and tumors (44, 179). For example, in ovarian carcinoma patients, a sizable fraction of PD-1^pos^ NK cells can be detected in PB, while larger percentages are present in the ascitic fluid of the same patients. Although PD-1 mRNA and protein are detectable at the cytoplasmic level, the mechanisms leading to its surface expression in human NK cells have not been defined yet. The detection of PD-1^pos^ in NK cells associated to tumors suggests that signals delivered by the tumor or its microenvironment may be involved in its surface expression. Besides PD-1, several other inhibitory checkpoints expressed by NK cells have been detected, and have been recently reviewed (180). Briefly, CD96 and TIGIT belong to the same family of the activating receptor DNAM-1 (their common ligands, CD155 and CD112, are nectin-like molecules). TIGIT was found to be up-regulated in tumor-associated NK cells in colorectal cancer. KLRG1 is expressed by NK cells upon activation (as well as by other lymphoid and myeloid cell types). LAG-3 marks exhausted T cells infiltrating tumors, while its possible modulatory effect on NK cells has not been defined yet. TIM-3 binds primarily to Galectin-9 but also to other tumor-associated antigens. A recently identified inhibitory receptor which is likely to play an important role in controlling NK cell activation, proliferation and function is IL-1R8, a member of IL-1 receptor family and a component of the human IL-37 receptor. Mice lacking IL1R8 do not develop carcinogen-induced hepatocarcinoma (181). Thus, it is conceivable that IL-1R8 blocking may unleash NK cells and promote efficient antitumor responses.

### PD-1/PD-L1 and Their Targeting in the Therapy of Cancer

As mentioned above, PD-1 may impair T cell responses against tumor cells. Importantly, immunotherapy with mAbs able to disrupt the PD-1/PD-L1 interaction proved to be highly effective, allowing unthinkable achievements in the cure of a fraction of tumor patients. In this context, the number of mAbs specific for PD-1 or PD-L1 approved for cancer therapy is growing fast and the spectrum of tumors for which the use of such agents is recommended includes non-small lung cancer, melanoma, urothelial cancers, kidney tumors, Hodgkin lymphoma, colorectal cancer, and hepatocellular carcinoma. While the use of such checkpoint inhibitors represents a real revolution in cancer treatment, in a still too large fraction of patients these agents are ineffective. Accordingly, a major goal is to predict the patient response to treatment, to avoid possible side effects and useless high costs of the therapy. The expression of PD-L1 on tumor cells undoubtedly represents a valuable biomarker. However, its predictive value is still unsatisfactory because of various limitations, including inter- and intra-tumor heterogeneous PD-L1 expression, use of PD-1/PD-L1 specific mAbs displaying a substantially different reactivity (182), different diagnostic material (biopsies vs. surgical specimens). Patient selection criteria are being standardized, for example, by defining the optimal number of biopsies that give results comparable to the whole tumor section, considered as the gold standard (183, 184). Given these limitations, research in progress is aimed at identifying additional checkpoints possibly to be used alone or in combination with PD-1/PD-L1 or CTLA-4.

### Therapeutic Use of Anti-KIR or Anti-NKG2A mAbs

After the launch of Innate-Pharma in Marseille, Alessandro's antibodies and his expertize were fundamental for the design of a program focalized on NK cells harnessing the blockade of inhibitory KIRs and NKG2A. Thus, three clinical grade mAbs were produced: Lirilumab (also called IPH2101, formerly 1-7F9) targeting KIRs, IPH4102 selectively recognizing KIR3DL2, and Monalizumab targeting NKG2A. These reagents are currently used in clinical trials to treat cancer patients. Thus, Lirilumab, a first-in-class humanized IgG4 mAb directed against a common epitope shared by KIR2D, disrupts the KIR/KIR-L interaction by rendering NK cells “alloreactive” and allows killing of tumor target cells. This antibody has been used in a phase I trial in association with Lenalidomide, for NK stimulation, to treat Multiple Myeloma. This combined treatment resulted to be safe, tolerable, and associated with signs of clinical efficacy (185). Because KIR3DL2 is highly expressed on cutaneous T cell lymphomas, including mycosis fungoides and Sézary syndrome (186), IPH4102 has been developed for the treatment of these diseases. Phase I trials provided evidence that the treatment is well-tolerated and efficacious. These encouraging results would support the large-scale clinical trials (187).

Recent studies identified NKG2A as an important checkpoint leading to remarkable antitumor effects in animal models (188–190). Encouraging results have also been obtained in a preliminary clinical trial in highly aggressive head and neck cancers (188). Notably, NKG2A, constitutively expressed by NK cells, can be induced also in T cells upon cytokine or antigen-induced activation (191, 192). Indeed, previous *in vitro* studies showed that mAb-mediated masking of NKG2A or KIRs, in both polyclonal and clonal NK or T cell populations, induced killing of HLA class I^pos^ tumor target cells (149, 188, 193–195). Vivier group also showed that the antitumor effects of anti-NKG2A mAb could be potentiated by the combined use of other therapeutic mAbs (188). Based on the expression of inhibitory checkpoint ligands on tumor cells, three therapeutic approaches have been proposed: (1) HLA-E^pos^ tumors lacking the expression of other ligands for inhibitory checkpoints or tumor antigens: blocking of NKG2A could unleash both NK- and T-cell-mediated antitumor responses in a murine model; (2) tumors expressing both HLA-E and PD-L1: by the combined blocking of NKG2A and PD-1/PD-L1 axis, not only enhancement of NK and T cell cytotoxicity but also induction of T cell proliferation and establishment of T cell memory were detected; (3) tumors expressing HLA-E and specific tumor antigens (e.g., EGF-R): blockade of NKG2A increased the efficacy of anti-EGF-R by allowing a CD16-mediated efficient ADCC by mature, highly cytotoxic NK cells.

## KIR and Haploidentical Hematopoietic Stem Cell Trasplantation

The discovery of both HLA class I specific inhibitory receptors and NK cell alloreactivity has been exploited, after a relatively short time interval, for the treatment of acute, high-risk leukemia in the haplo-HSCT setting. HSCT is the life-saving therapy for such leukemia with adverse molecular or cytogenetic characteristics, or with poor response to chemotherapy, or relapsing. However, an HLA 10/10 allelic matched donor, whether sibling or unrelated, is available only for two out of three patients, and this proportion can be even lower for patients belonging to certain ethnic groups. Thus, haplo-HSCT has been developed in the attempt to rescue these patients (196). In such transplantation setting, donor derived NK cells are defined as “alloreactive” when they express, as inhibitory receptor, exclusively KIR(s) specific for self-HLA class I allele(s) (KIR-L), allowing the elimination of patient cells missing that particular KIR-L. A seminal study by Ruggeri et al. (197) reported a 5-year survival probability of ~60% in adult AML patients in case of donor NK alloreactivity (KIR/KIR-L mismatch in graft vs. host direction), while survival was <5% in its absence. In 2002, Alessandro and some authors of the present contribution started a fruitful collaboration with Franco Locatelli, first at the University of Pavia and subsequently at the Ospedale Pediatrico Bambino Gesù in Roma (149, 198). In these studies, patients were represented by children and young adults (1–20 years) with acute high-risk ALL or AML. Notably, in the haplo-HSCT setting, most pediatric patients find an available donor, represented by one parent.

### KIR-Based Donor Selection

In view of the major relevance of NK alloreactivity, a great attention was paid by our group to the selection of the best possible donor, based primarily on the “perfect mismatch” (199) and on the size of alloreactive NK cell subset. To this end, a reliable method was developed to define the presence and the frequency of such subpopulation in potential donors (149). This is based on combined genotypic and phenotypic analyses to assess (i) the presence in the donor of KIR-L(s) absent in the recipient, (ii) donor's *KIR* genotype to evaluate the presence of KIR(s) specific for the mismatched KIR-L(s), and (iii) identification of the alloreactive subset using appropriate anti-KIR and anti-NKG2A mAb combinations (198). Leukemia patients, after the conditioning regimen, receive very high numbers (“megadoses”) of highly purified T-depleted CD34^pos^ cells, isolated either from bone marrow (BM) or PB. Isolation from PB is preceded by donor treatment with G-CSF in order to “mobilize” CD34^pos^ cells from BM. Notably, different from AML, adult high-risk ALL were poorly responding to haplo-HSCT even in the presence of donor NK alloreactivity. Thus, it was somewhat surprising to find opposite results in pediatric patients. Indeed, in case of NK alloreactivity, the survival rate was ~70% in ALL and ~40% in AML, while in the absence of NK alloreactivity, the percentages were ~35 and ~20%, respectively. Of note, in the transplantation setting with “pure CD34^pos^” cells, most deaths due to leukemia relapses or transplant-related mortality occurred early, within few weeks/months after transplantation. As mentioned above, NK cells, during their differentiation from HSC, first express CD94:NKG2A, while KIR are acquired only at later stages. Accordingly, the generation of alloreactive, mature NK cells requires a relatively long time interval (6–8 weeks). Thus, for several weeks, the absence of mature (alloreactive) NK cells together with the absence of adaptive immunity may be critical for the control of leukemia relapses and infections. The survival rate of leukemia patients receiving a CD34^pos^ haplo-HSC could be considered satisfactory in view of the extremely poor prognosis of patients not receiving transplantation. However, the delayed appearance of alloreactive NK cells, conceivably explaining the occurrence of early relapses and infections, suggested, in 2010, the application of a new graft manipulation based on the depletion of αβ T lymphocytes and CD19^pos^ B lymphocytes. Thanks to this novel approach, the graft infused contains, besides HSC, other cell types, including intermediate precursors (CD34^neg^), various myeloid cell types, mature NK cells, and γδ T lymphocytes. Notably, both NK and γδ T lymphocytes may exert a potent anti-leukemia activity and contribute to the control of infections. Accordingly, patients could immediately benefit of high numbers (30–40 × 10^6^/kg) of donor mature NK cells and γδ T cells (3–5 × 10^6^/kg). The clinical outcome in 80 children was excellent with an overall survival of ~70% not only in ALL, but also in AML (200). Surprisingly, NK alloreactivity did not appear to play a relevant role in the clinical outcome. The marked improvement of the survival rate in the absence of alloreactivity may be due, at least in part, to the additional selection criteria progressively applied to identify the most suitable among the available donors. These include the following positive characteristics: (i) *KIR* B/X genotype, (ii) HLA-C1^pos^/KIR2DS1^pos^ for HLA-C2^pos^ recipients, (iii) high absolute cell numbers of NK and γδT lymphocytes, and (iv) high expression of NKp46 and presence of NKG2C (198, 200). The presence of mature NK and γδ T from the beginning of the transplantation and their persistence in the recipient as efficient immune cells, due to the lack of GvHD prophylaxis, can explain the successful clinical results. In immunocompromised patients undergoing allogeneic HSCT, HCMV infection/reactivation frequently occurs (201). Several studies documented a role of HCMV in promoting rapid NK cell maturation and in the selective expansion of NKG2C^pos^ adaptive NK cells (34, 202, 203). This HCMV related effect on NK cell repertoire was also observed in pediatric patients receiving αβ T/B cell-depleted haplo-HSCT (204). Remarkably, a study revealed that, in NKG2C^neg/neg^ umbilical cord blood donors, HCMV infection promoted a maturation and expansion of NK cells expressing aKIRs (205). These findings suggest that, in the absence of NKG2C, aKIRs may sense HCMV infection and promote selective expansion of mature aKIR^pos^ NK cells, underscoring the concept that aKIRs may sense microbial infections.

Taken together, the clinical results in haplo-HSCT indicate that cells belonging to innate immunity play a crucial role in the cure of patients with otherwise lethal leukemia and provide a solid background to further improve the survival rate and to implement novel NK-based therapies (e.g., CAR-NK). In this context, helper ILC, primarily ILC-3, may provide a tool to further improve early innate defense against pathogens after HSCT. Notably, it has been shown that ILC-3 development may be favored by given sources of HSC for transplantation (206).

## Concluding Remarks

There is little doubt that major advances in immunology and the exploitation of fundamental discoveries offered invaluable tools for the therapy of cancer. Indeed, the recent years witnessed unprecedented successes in the cure of different, high aggressive tumors and leukemia. In this context, NK cells and their receptors were shown to play an unexpected role. Thus, the discovery of KIRs provided the molecular basis for haplo-HSCT, which allowed the cure of patients with acute, high-risk leukemia for whom no HLA-compatible donors were available. These data also provided the first evidence that not only T and B lymphocytes, but also cells of the innate immunity may be exploited for tumor therapy. While checkpoint inhibitors targeting PD-1 or CTLA-4 represent the most effective tools to successfully treat a fraction of otherwise incurable cancer patients, their combined use with therapeutic antibodies blocking NKG2A and KIR may further extend the benefit to a large proportion of patients. Thus, the Alessandro's legacy, besides unraveling the molecular mechanisms of NK cell function, also extends to thousands of leukemia patients recovered from their otherwise fatal disease.

The continuous interest and evolution on KIR research is also witnessed by the organization of a KIR Workshop taking place every year and a half, allowing to share the most recent advances on KIRs in health and disease, with a fruitful exchange of ideas and knowledge in a friendly atmosphere. Last October, the KIR Workshop 2018 has been organized by our group, and Alessandro had begun to collaborate with us on its organization and realization. This meeting was dedicated to Alessandro.

## Author Contributions

LM, DP, and MF wrote, referenced the review, and prepared the figures. All authors contributed with their relevant published studies, cited in this review, and critically revised the manuscript.

### Conflict of Interest Statement

The authors declare that the research was conducted in the absence of any commercial or financial relationships that could be construed as a potential conflict of interest.
